# Supplementary figures and images for: IGF1R Deficiency Modulates Brain Signaling Pathways and Disturbs Mitochondria and Redox Homeostasis
Source: Biomedicines. 2021 Feb 6;9(2):158. doi: 10.3390/biomedicines9020158 (PMC7915200; doi:10.3390/biomedicines9020158)

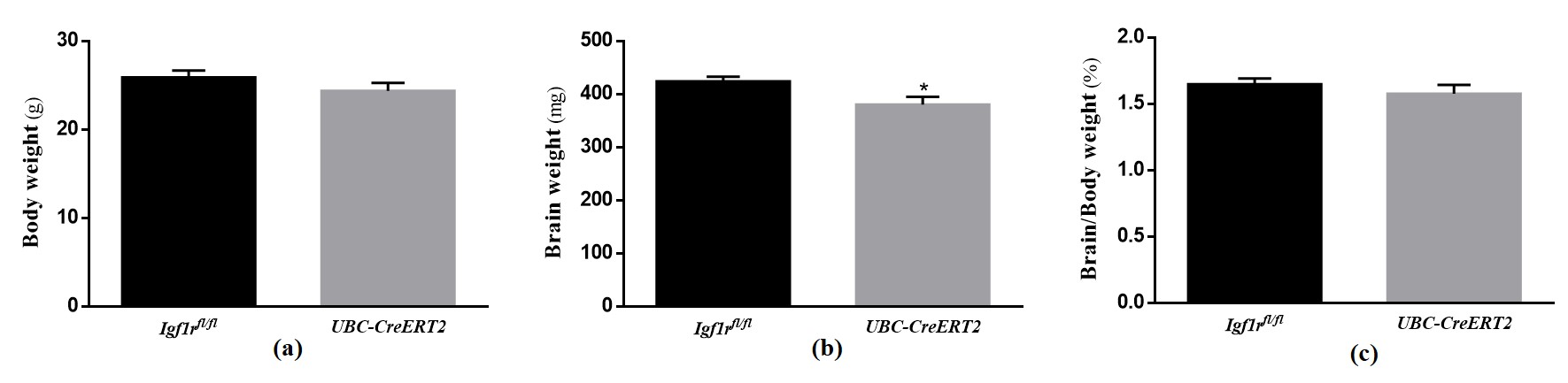

Supplement: Supplementary file 1 [file biomedicines-09-00158-s001.zip › Supplementary data_uncropped WBs/Figure S1.jpg]

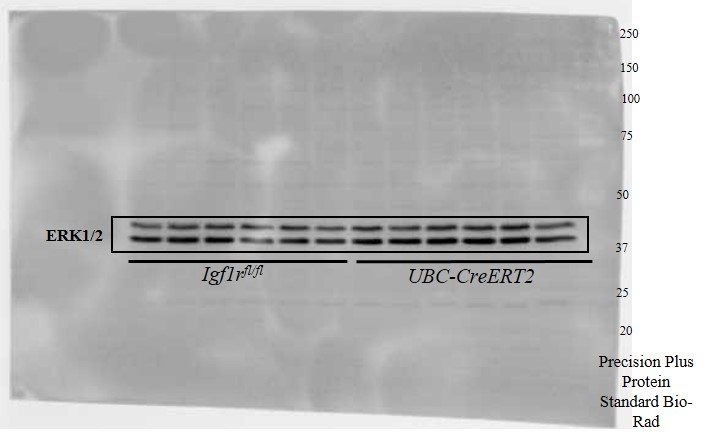

Supplement: Supplementary file 1 [file biomedicines-09-00158-s001.zip › Supplementary data_uncropped WBs/Figure S10.jpg]

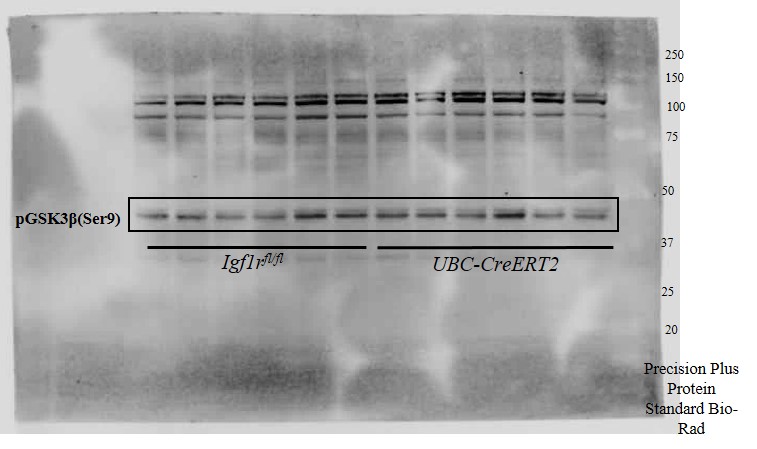

Supplement: Supplementary file 1 [file biomedicines-09-00158-s001.zip › Supplementary data_uncropped WBs/Figure S11.jpg]

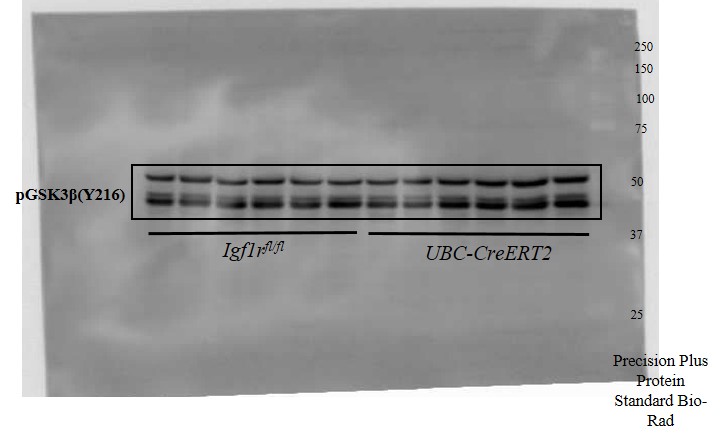

Supplement: Supplementary file 1 [file biomedicines-09-00158-s001.zip › Supplementary data_uncropped WBs/Figure S12.jpg]

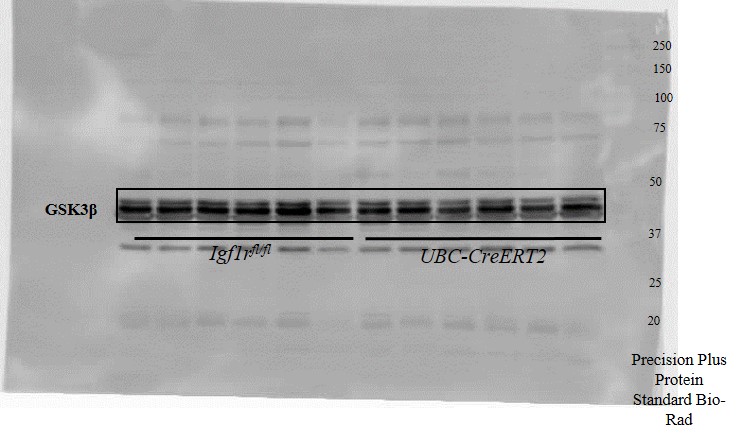

Supplement: Supplementary file 1 [file biomedicines-09-00158-s001.zip › Supplementary data_uncropped WBs/Figure S13.jpg]

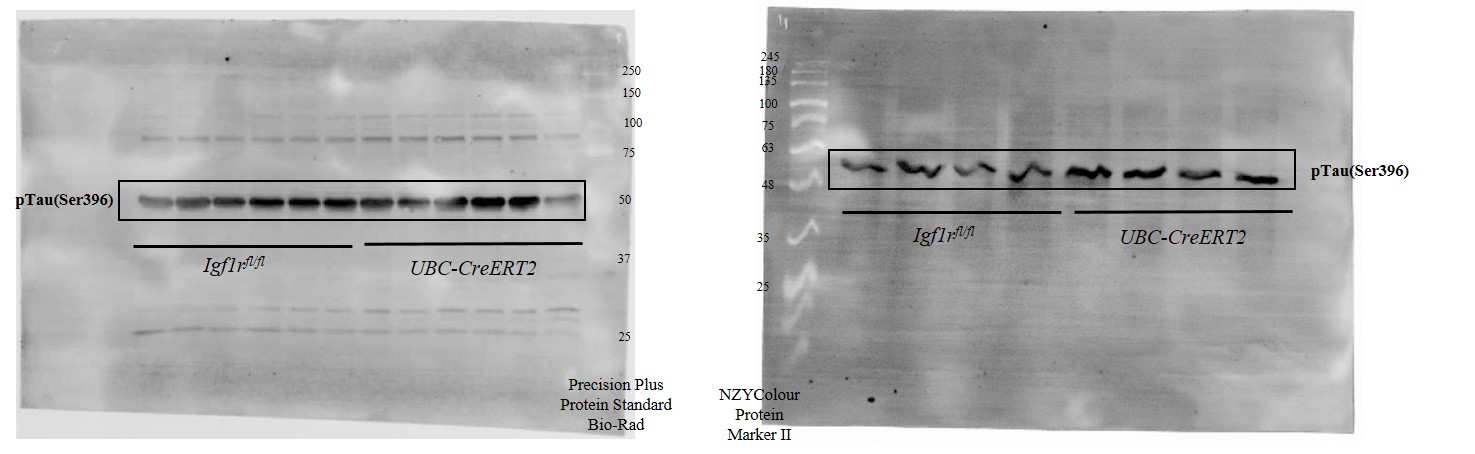

Supplement: Supplementary file 1 [file biomedicines-09-00158-s001.zip › Supplementary data_uncropped WBs/Figure S14.jpg]

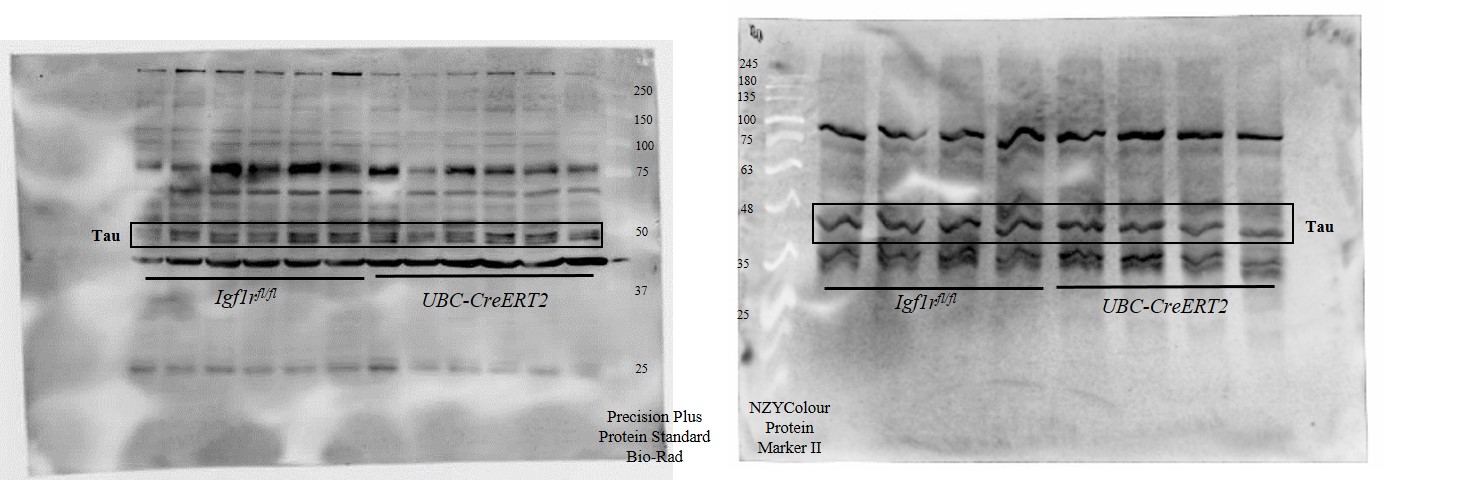

Supplement: Supplementary file 1 [file biomedicines-09-00158-s001.zip › Supplementary data_uncropped WBs/Figure S15.jpg]

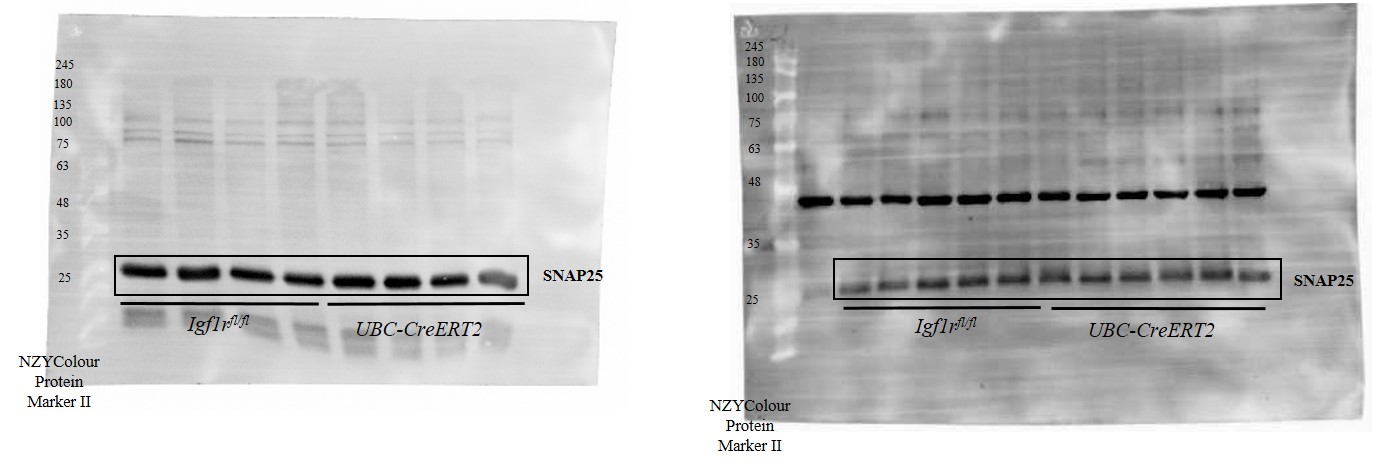

Supplement: Supplementary file 1 [file biomedicines-09-00158-s001.zip › Supplementary data_uncropped WBs/Figure S16.jpg]

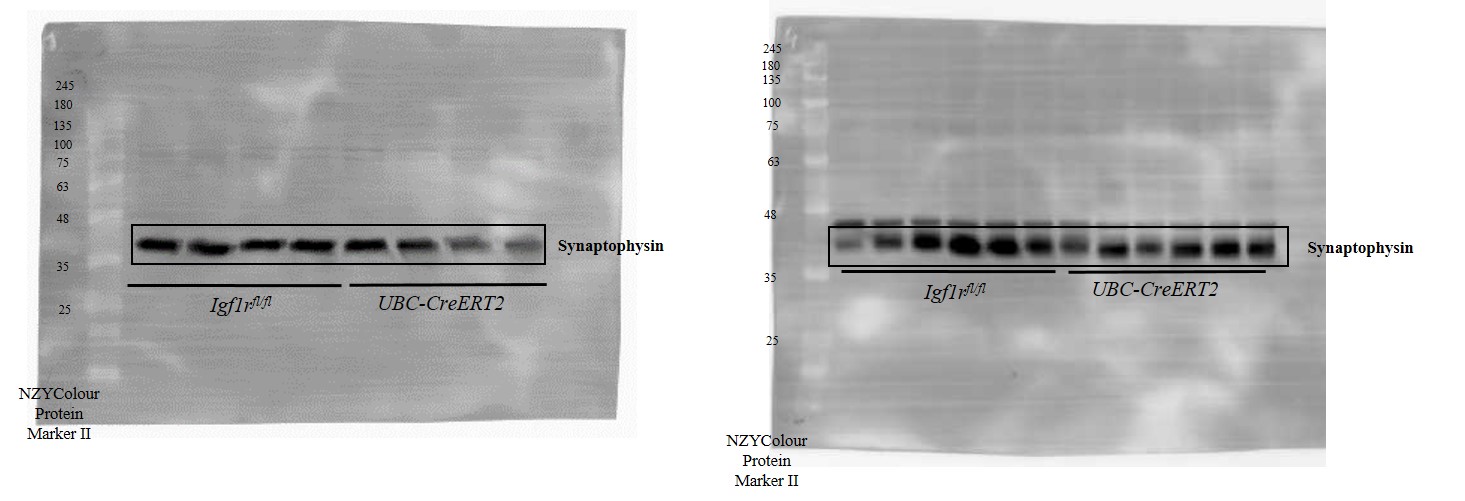

Supplement: Supplementary file 1 [file biomedicines-09-00158-s001.zip › Supplementary data_uncropped WBs/Figure S17.jpg]

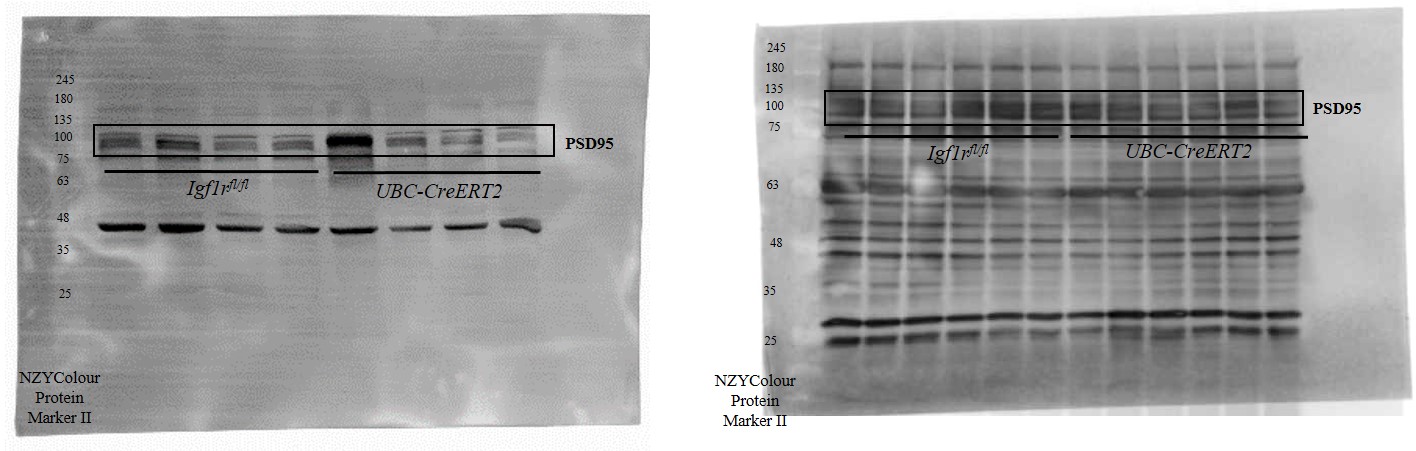

Supplement: Supplementary file 1 [file biomedicines-09-00158-s001.zip › Supplementary data_uncropped WBs/Figure S18.jpg]

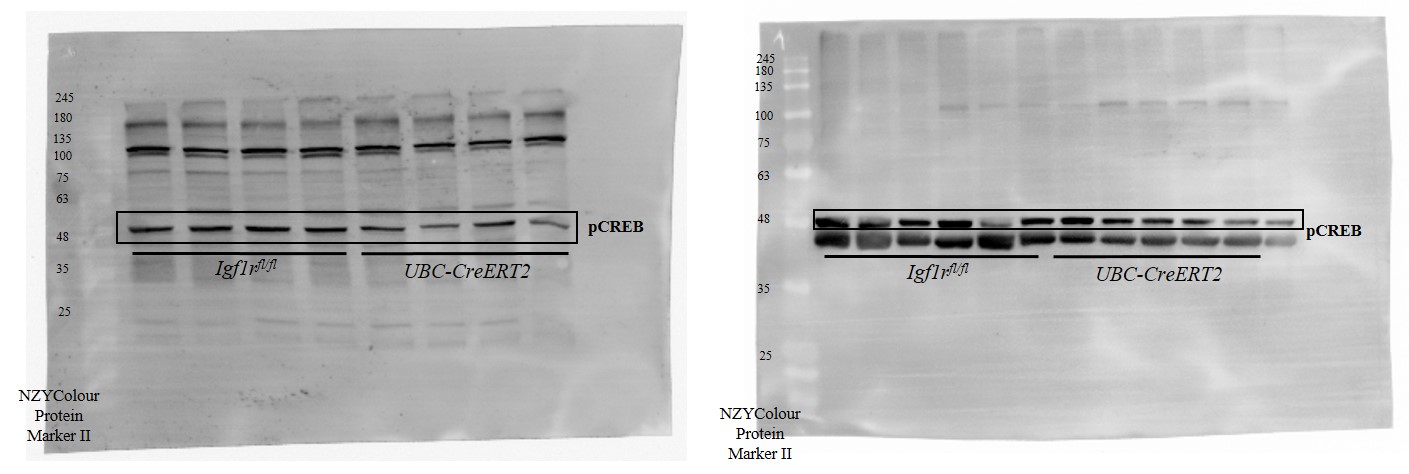

Supplement: Supplementary file 1 [file biomedicines-09-00158-s001.zip › Supplementary data_uncropped WBs/Figure S19.jpg]

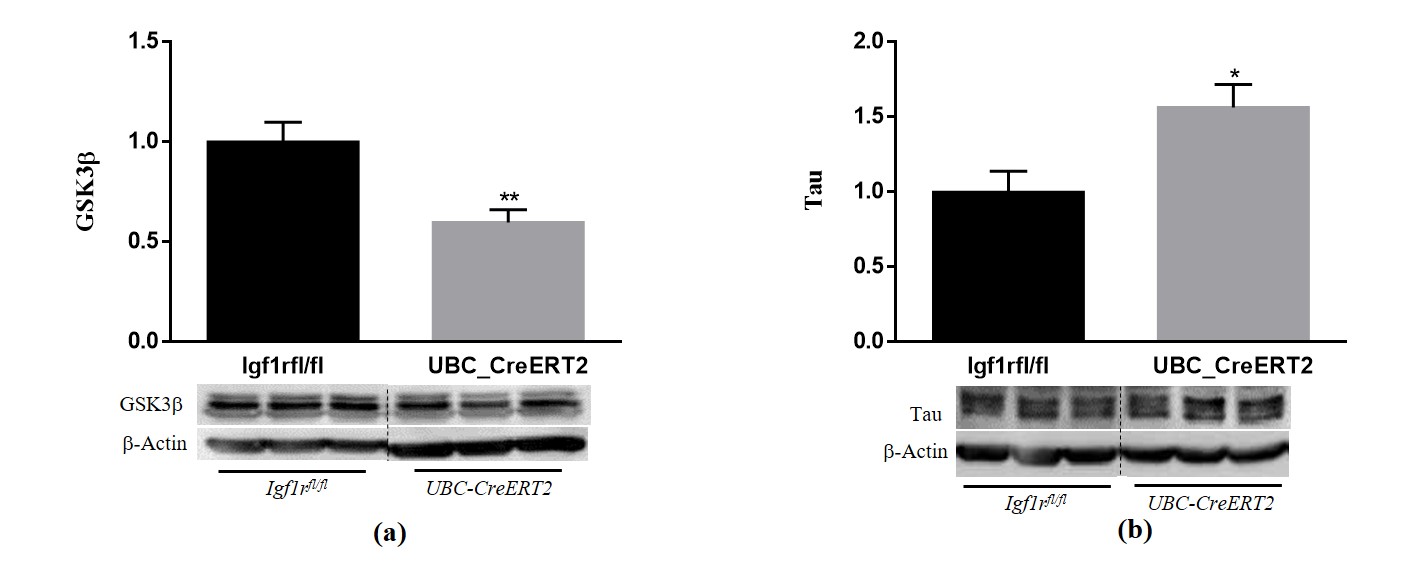

Supplement: Supplementary file 1 [file biomedicines-09-00158-s001.zip › Supplementary data_uncropped WBs/Figure S2.jpg]

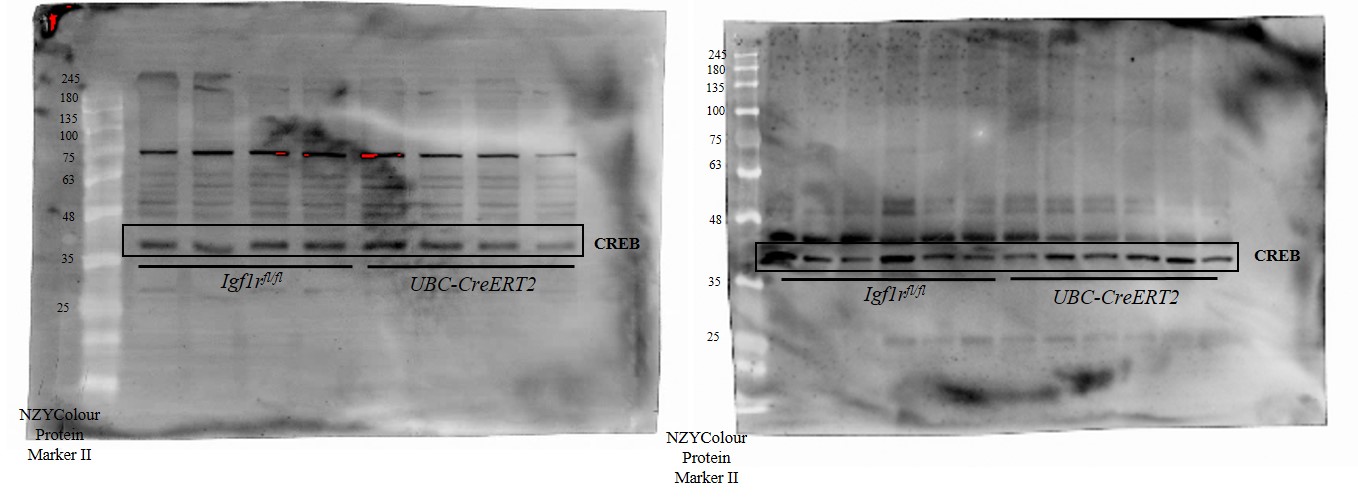

Supplement: Supplementary file 1 [file biomedicines-09-00158-s001.zip › Supplementary data_uncropped WBs/Figure S20.jpg]

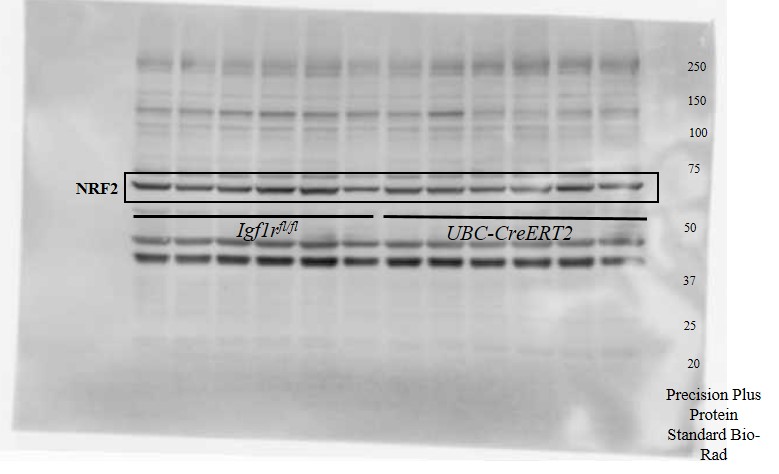

Supplement: Supplementary file 1 [file biomedicines-09-00158-s001.zip › Supplementary data_uncropped WBs/Figure S21.jpg]

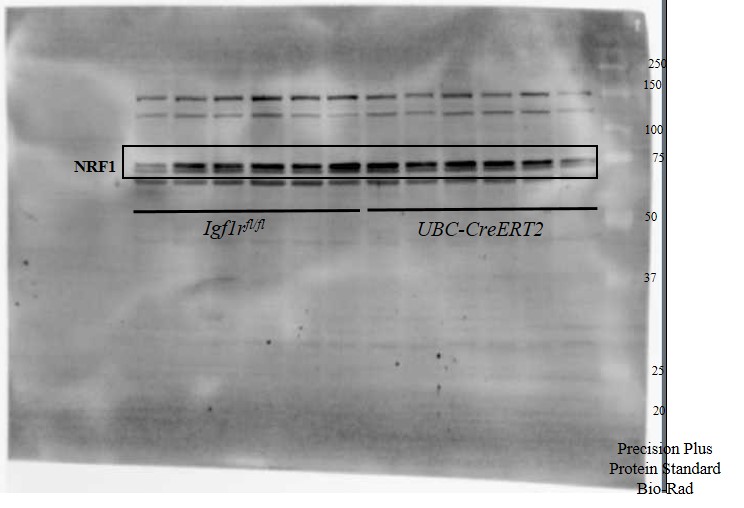

Supplement: Supplementary file 1 [file biomedicines-09-00158-s001.zip › Supplementary data_uncropped WBs/Figure S22.jpg]

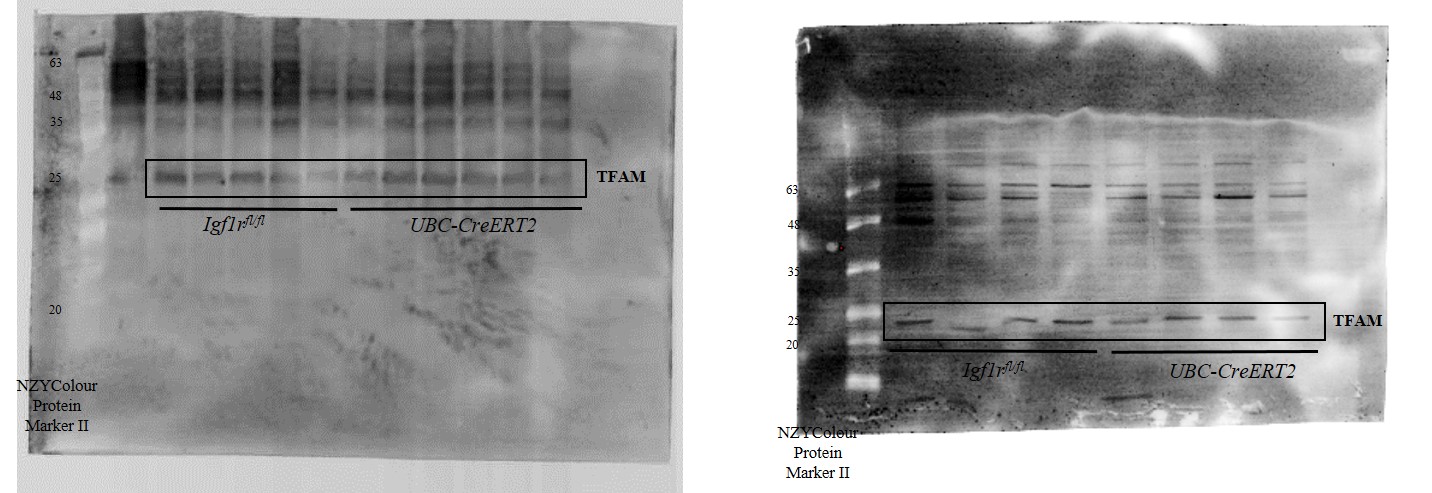

Supplement: Supplementary file 1 [file biomedicines-09-00158-s001.zip › Supplementary data_uncropped WBs/Figure S23.jpg]

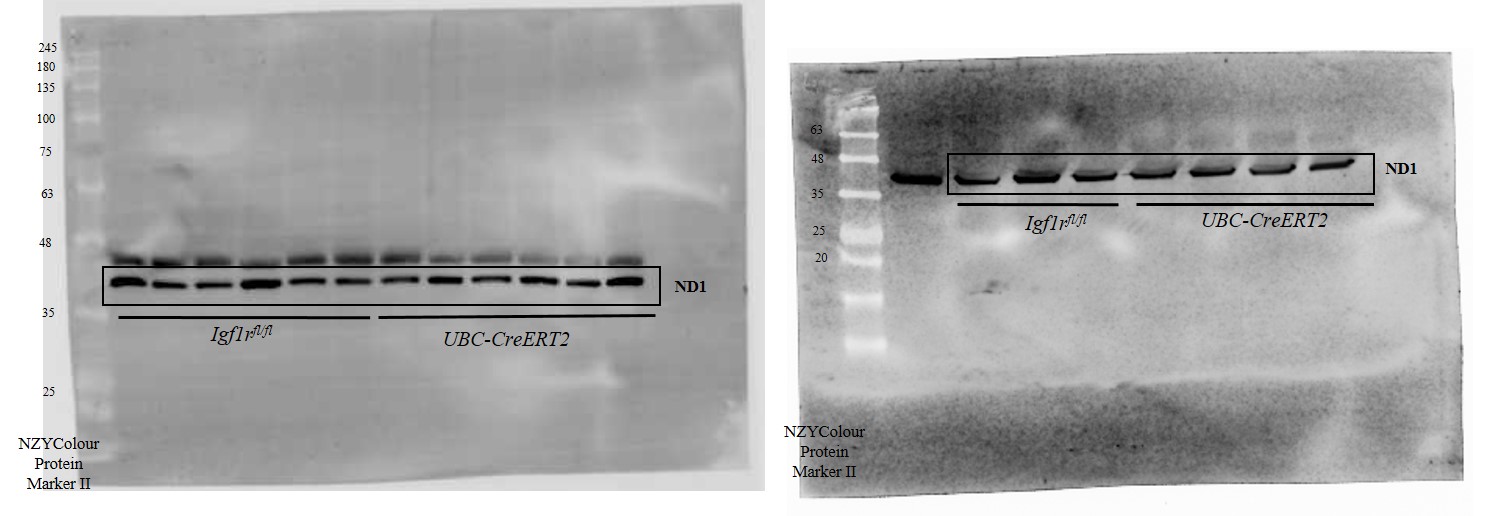

Supplement: Supplementary file 1 [file biomedicines-09-00158-s001.zip › Supplementary data_uncropped WBs/Figure S24.jpg]

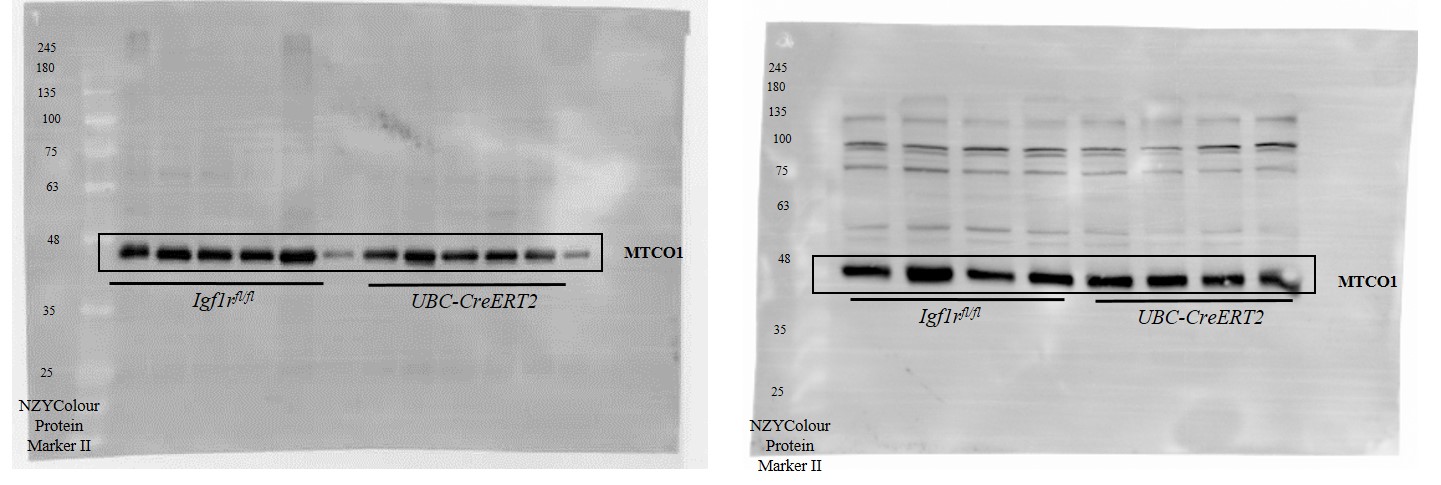

Supplement: Supplementary file 1 [file biomedicines-09-00158-s001.zip › Supplementary data_uncropped WBs/Figure S25.jpg]

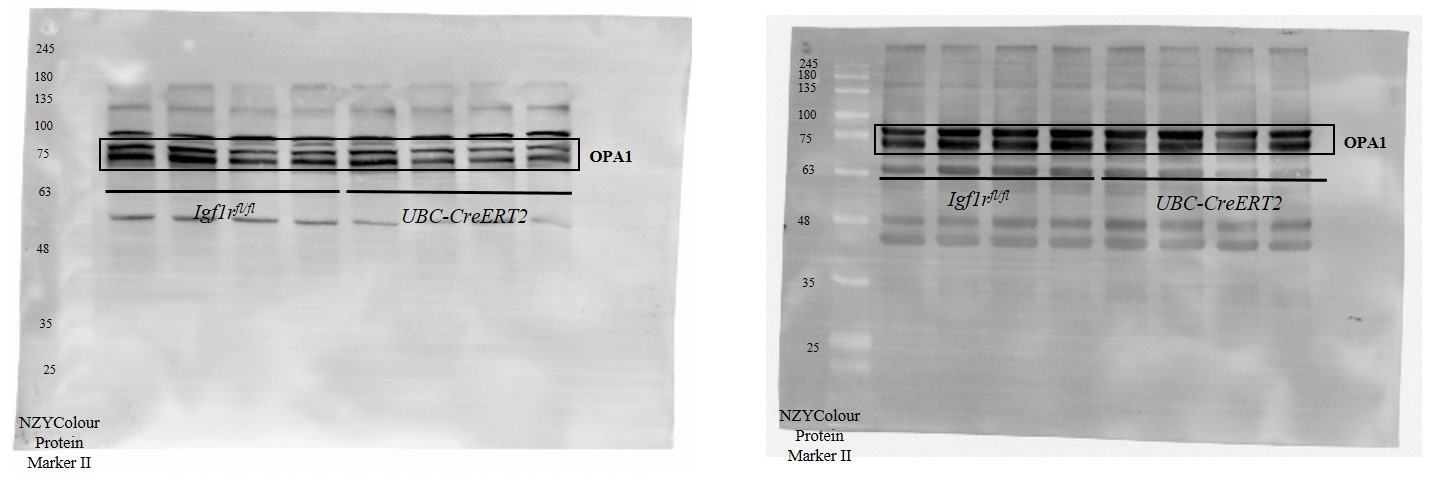

Supplement: Supplementary file 1 [file biomedicines-09-00158-s001.zip › Supplementary data_uncropped WBs/Figure S26.jpg]

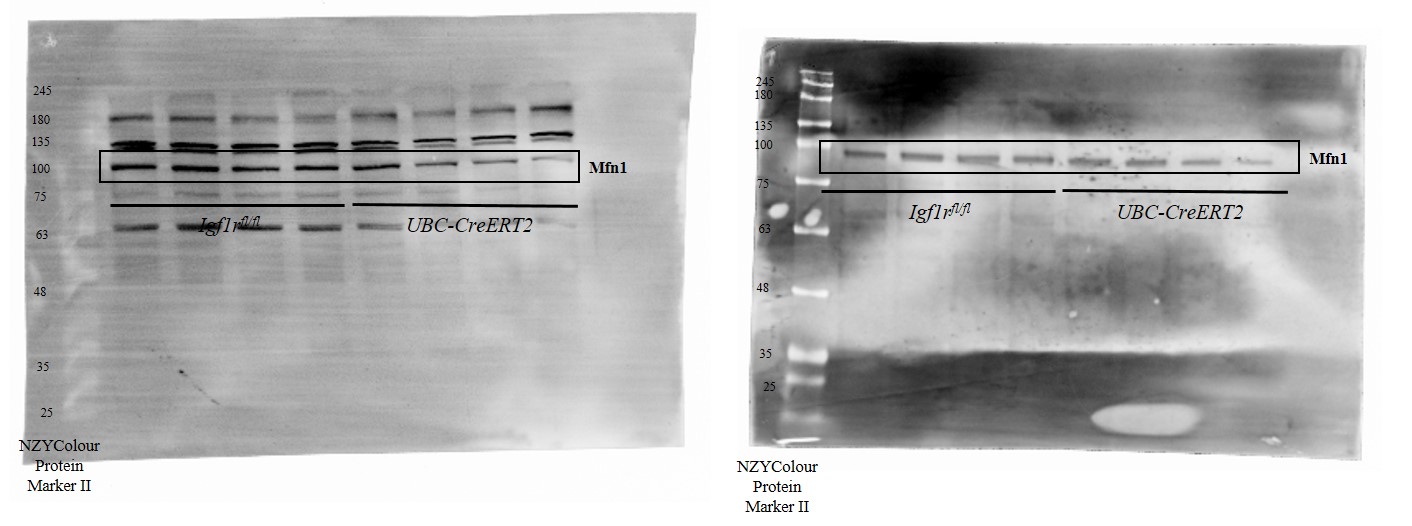

Supplement: Supplementary file 1 [file biomedicines-09-00158-s001.zip › Supplementary data_uncropped WBs/Figure S27.jpg]

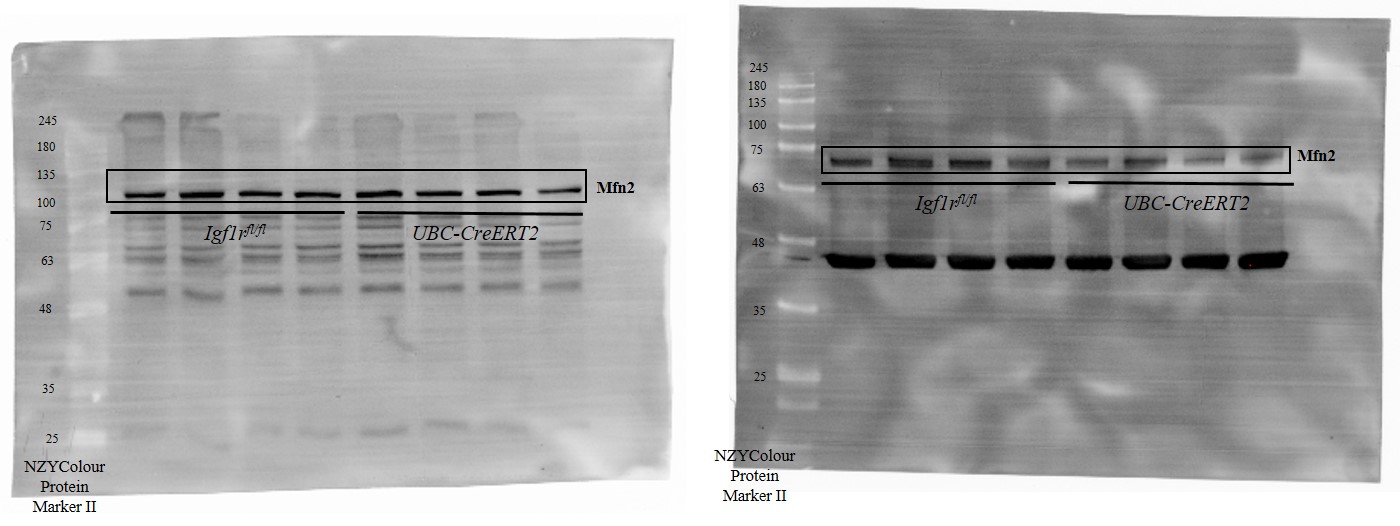

Supplement: Supplementary file 1 [file biomedicines-09-00158-s001.zip › Supplementary data_uncropped WBs/Figure S28.jpg]

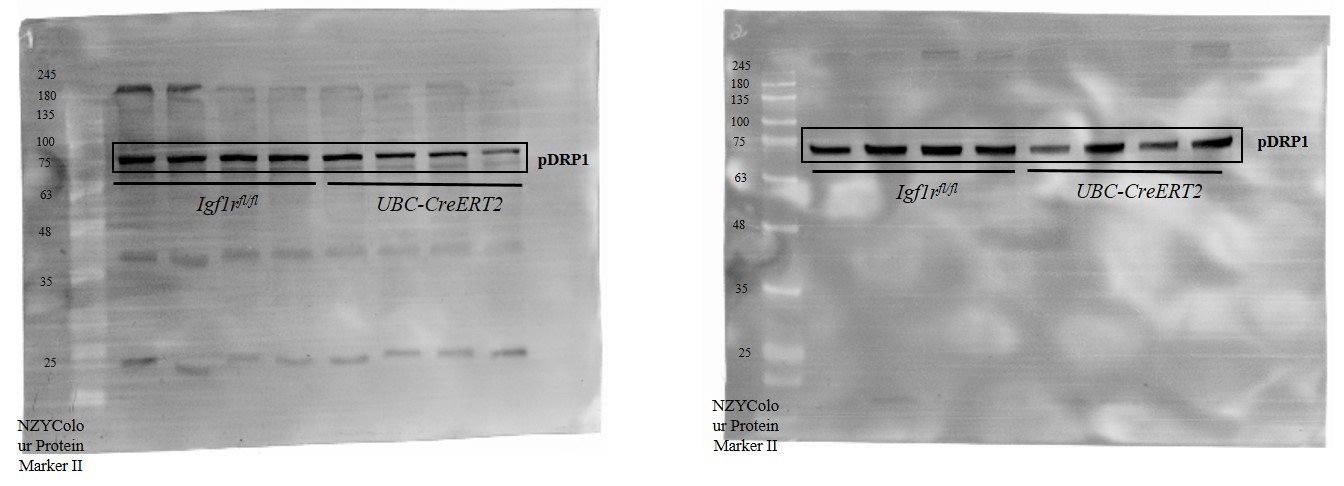

Supplement: Supplementary file 1 [file biomedicines-09-00158-s001.zip › Supplementary data_uncropped WBs/Figure S29.jpg]

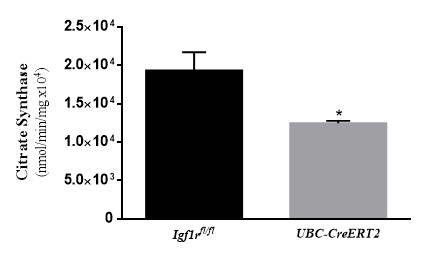

Supplement: Supplementary file 1 [file biomedicines-09-00158-s001.zip › Supplementary data_uncropped WBs/Figure S3.jpg]

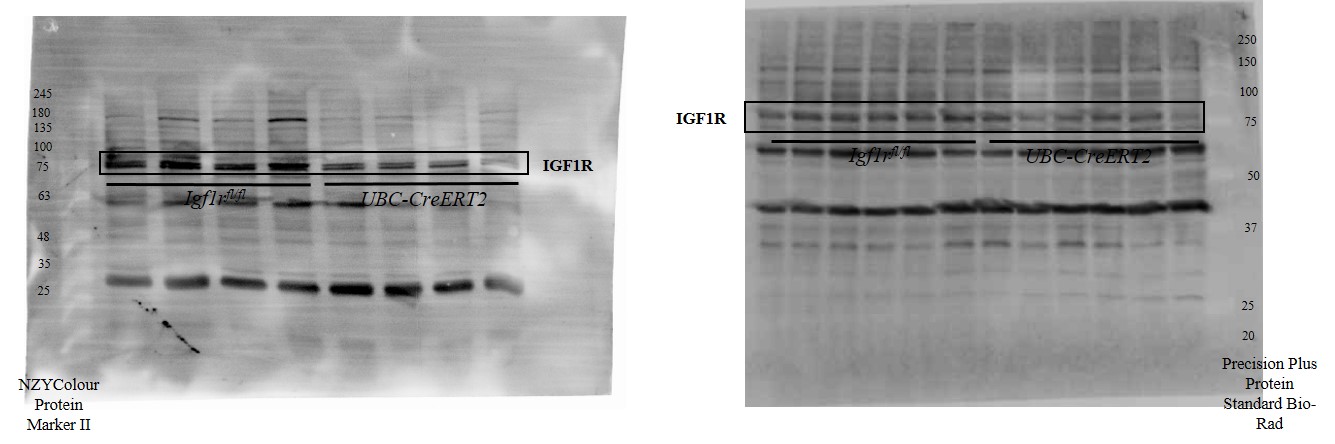

Supplement: Supplementary file 1 [file biomedicines-09-00158-s001.zip › Supplementary data_uncropped WBs/Figure S4.jpg]

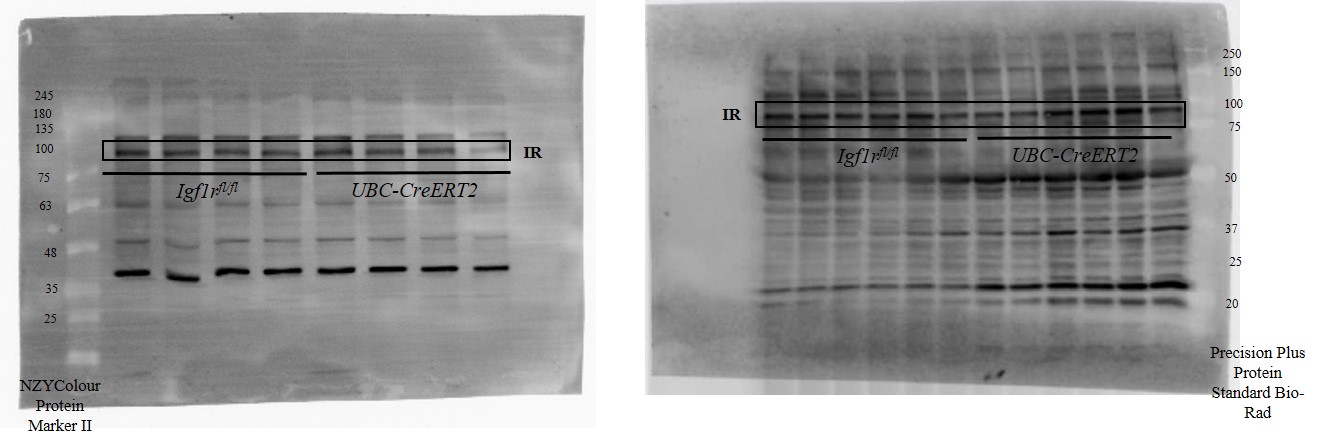

Supplement: Supplementary file 1 [file biomedicines-09-00158-s001.zip › Supplementary data_uncropped WBs/Figure S5.jpg]

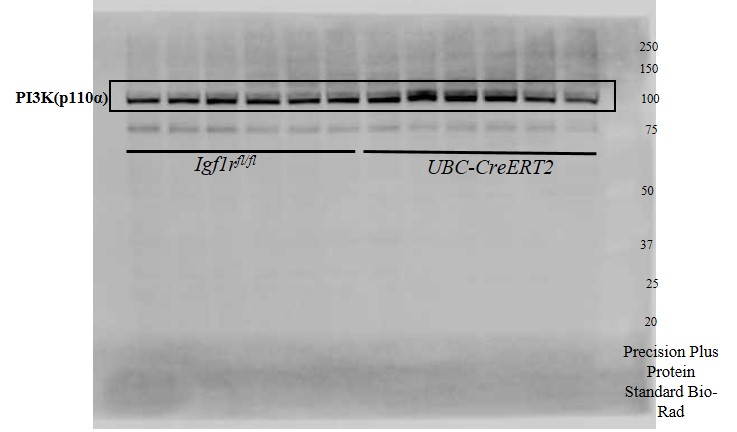

Supplement: Supplementary file 1 [file biomedicines-09-00158-s001.zip › Supplementary data_uncropped WBs/Figure S6.jpg]

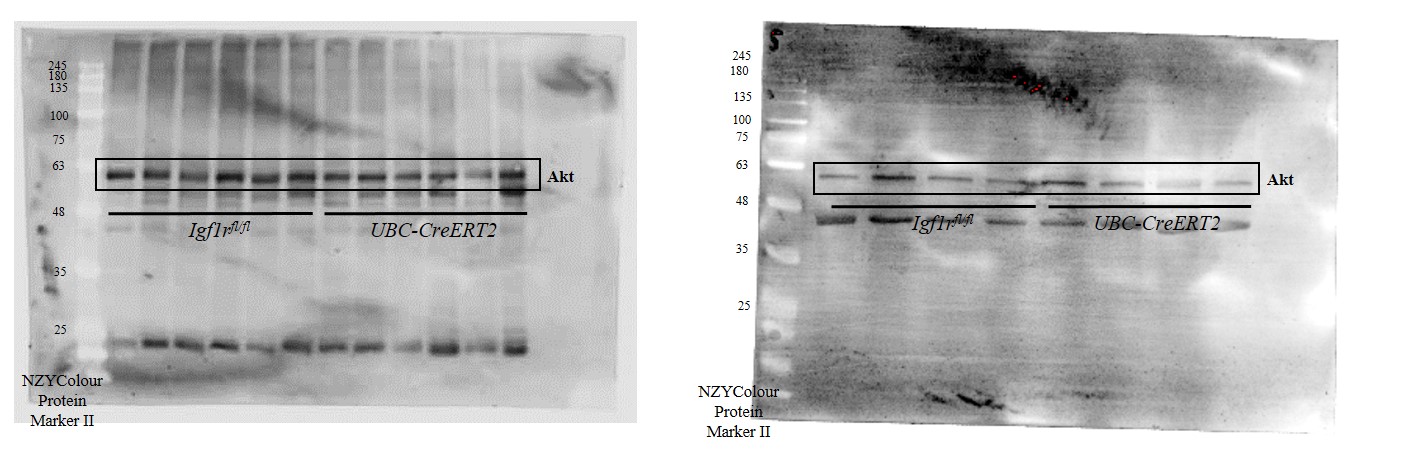

Supplement: Supplementary file 1 [file biomedicines-09-00158-s001.zip › Supplementary data_uncropped WBs/Figure S7.jpg]

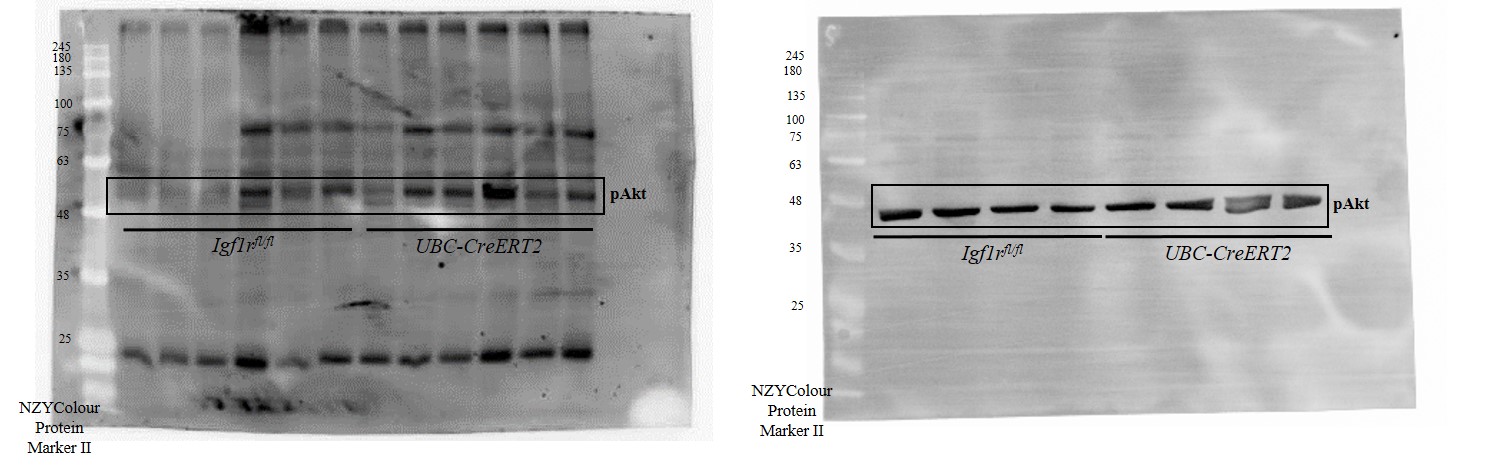

Supplement: Supplementary file 1 [file biomedicines-09-00158-s001.zip › Supplementary data_uncropped WBs/Figure S8.jpg]

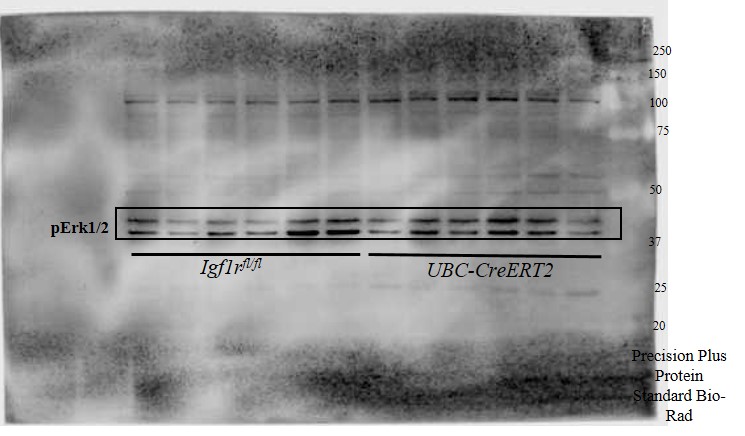

Supplement: Supplementary file 1 [file biomedicines-09-00158-s001.zip › Supplementary data_uncropped WBs/Figure S9.jpg]
